# Anticoccidial activity of a botanical natural product based on eucalyptus, apigenin and eugenol against *Eimeria tenella* in broiler chickens

**DOI:** 10.1186/s13071-024-06409-z

**Published:** 2024-08-02

**Authors:** Tiantian Geng, Xiaodie Ruan, Ying Xie, Bang Shen, Rui Fang, Junlong Zhao, Yanqin Zhou

**Affiliations:** 1 Key Laboratory Preventive Veterinary of Hubei Province, College of Veterinary Medicine, Huazhong Agricultural University, Wuhan, 430070 Hubei People’s Republic of China; 2 State Key Laboratory of Agricultural Microbiology, Huazhong Agricultural University, Wuhan, 430070 Hubei People’s Republic of China; 3 Shanchuan Biotechnology (Wuhan) Co., Ltd., Wuhan, People’s Republic of China

**Keywords:** Coccidiosis, *Eimeria tenella*, Botanical natural product formulation, Safety test, Broilers

## Abstract

**Background:**

Chicken coccidiosis is an intracellular parasitic disease that presents major challenges to the development of the commercial poultry industry. Perennial drug selective pressure has led to the multi-drug resistance of chicken coccidia, which makes the prevention and control of chicken coccidiosis extremely difficult. In recent years, natural plant products have attracted the attention of researchers due to their inherent advantages, such as the absence of veterinary drug residues. The development of these natural products provides a new direction for the prevention and treatment of chicken coccidiosis.

**Methods:**

The anticoccidial effect of a natural plant product combination formulation (eucalyptus oil + apigenin + eugenol essential oil) was tested against *Eimeria tenella* in broilers. To search for the optimal concentration of the combination formulation, we screened 120 broilers in a chicken cage trial in which 100 broilers were infected with 5 × 10^4^ sporulated *Eimeria tenella* oocysts; broilers receiving a decoquinate solution was set up as a chemical control. The optimal anticoccidial concentration was determined by calculating the anticoccidial index (ACI), and the suitable concentration was used as the recommended dose for a series of safety dose assessment tests, such as feed conversion ratio (FCR), hematological indices and serum biochemical indices, as well as liver and kidney sections, at onefold (low dose), threefold (medium dose) and sixfold (high dose) the recommended dose (RD).

**Results:**

The results showed that this combination formulation of three plant natural products had a better anticoccidial effect than formulations containing two plant natural products or a single one, with an ACI of 169.3. The dose gradient anticoccidial test revealed that the high-dose formulation group had a better anticoccidial effect (ACI = 169.2) than the medium- and low-dose groups. The safety evaluation test showed that concentrations of the formulation at one-, three- and sixfold the RD were non-toxic to Arbor Acres broilers, indicating the high safety of the combination formulation.

**Conclusions:**

The combination formulation showed not only a moderate anticoccidial effect but also had a high safety profile for broilers. The results of this study indicate a new alternative for the prevention and control of coccidiosis in broilers.

**Graphical Abstract:**

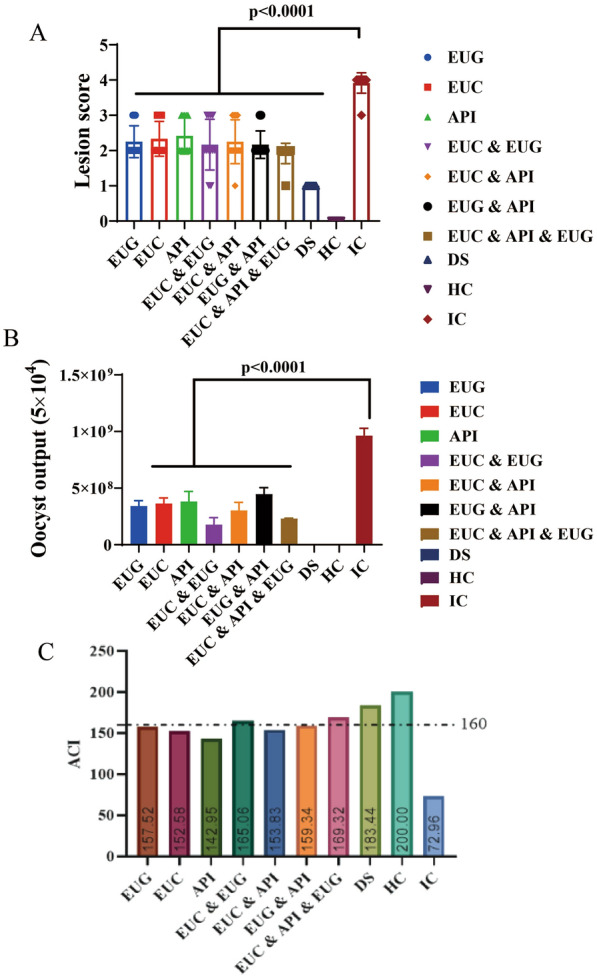

**Supplementary Information:**

The online version contains supplementary material available at 10.1186/s13071-024-06409-z.

## Background

Chicken coccidiosis is an intracellular parasitic disease that seriously endangers the health of poultry [1–3]. There are seven common pathogens of chicken coccidiosis [4], among which *Eimeria tenella* is the most widely distributed and the most damaging species and, consequently, this species is often used as a model pathogenic species for researchers to study chicken coccidiosis [5]. *Eimeria tenella* often parasitizes the cecal tissue of poultry, subsequently completing its life history of sexual reproduction and asexual reproduction in the intestinal epithelial cells. It usually causes extensive bleeding and spots in the cecal mucosa, and thickening and swelling of the intestinal wall to three- to fivefold the original size. The intestinal contents are often white cheese-like material or dark-red intestinal core [6]. Chicken coccidia have a very complex life history, which makes the prevention and control of the disease difficult [7]. Unsporulated oocysts develop into sporulated oocysts under suitable temperature and humidity conditions [8]. Chicken are infected with coccidiosis by ingesting feed or drinking water contaminated with sporulated oocysts. The disease will cause an expulsion of bloody stools by the host, and can even cause death in severe cases [9].

At present, the main method to prevent and control chicken coccidiosis is to add anticoccidial drugs to feed. However, veterinary drug residues pose a serious threat to the health of consumers [10]. Many alternative solutions have been proposed to solve this problem, including vaccines and the rotation of common chemicals every 3e months [11], but the risk of veterinary drug residues remains. New vaccines are constantly being developed, but it takes a long time from the development of new vaccine and their actual application in the real world [12]. In recent years, some researchers have proposed the development of a plant natural product with anticoccidial effects [13, 14] to solve the veterinary drug residue problem while also alleviating drug resistance problem, and plant natural product development has gradually become a new research hot topic for the prevention and control of chicken coccidiosis[15, 16]. Wang et al. found that artemisinin (effective concentration of 48.62 mg/kg) has a moderate anticoccidial effect, with an anticoccidial index (ACI) of 144.05 [17]. Chang et al. reported that a medium dose of garlic essential oil (0.06 ml/l) exhibits a desirable anticoccidial effect, with an ACI of 167.57 [18]. It is also considered that combinations of plant natural products may have even better anticoccidial effects. For example, Felici et al. [19] studied a combination of theymol, carvacrol and saponins and found that this combination had in vitro inhibitory activity against coccidia in chickens. Although this study [19] was an exploration of plant natural product combinations for in vitro anticoccidial activity, the results open the door to the potential applicability of plant natural combinations for the prevention and control of chicken coccidiosis.

 Eugenol essential oil is known to be an extremely safe essential oil and is recognized as a non-mutagenic and non-carcinogenic agent by the U.S. Food and Drug Administration (FDA) [20]. Eucalyptus oil is a common antimicrobial, antioxidant essential oil which also has nematicidal properties [21]. Apigenin is an edible plant-derived flavonoid that has been reported to be an anti-cancer agent [22]. In the present study, we combined these three plant natural products to form a combination of plant natural products that may have anticoccidial effects. Therefore, the purpose of this study was to investigate this combination formulation of multiple plant natural products and its anticoccidial effect and safety in chicken cage experiments. This study provides data to support the development of a plant natural product combination with anticoccidial effects.

## Methods

### Drugs and essential oils

Eugenol essential oil (batch number: 20230710) and eucalyptus oil (batch number: 20231025) were obtained from Shanchuan Biotechnology Co., Ltd. (Wuhan, China). Apigenin (batch number: D220833) was obtained from Yuanye Biotechnology Co., Ltd. (Shanghai, China), and the decoquinate solution (batch number: 20210826) was obtained from Luxi Veterinary Drug Co., Ltd., (Shandong, China).

### Ethics statement

All animal experiments were approved by the Ethics Committee of Huazhong Agricultural University (Approval Number: HZAUCH-2024-0004). All of the animal experiments were conducted in strict accordance with the National Institutes of Health (NIH) Guidelines for the Care and Use of Laboratory Animals [23].

### Study design

#### *Eimeria tenella* oocysts (Xiantao strain)

*Eimeria tenella* Xiantao strain that was used in this study was stored in the Parasite laboratory of Huazhong Agricultural University. The *Eimeria tenella* used in the experiments was passaged every 6 months to maintain parasite viability.

#### Broilers

A total of 350 0-day-old Arbor Acres broilers were purchased from Zhengkang Livestock and Poultry Co., Ltd (Jingzhou city, China). All chickens were kept in coccidia-free cages (0.7 × 0.7 × 0.4 m) with ad libitum access to food and water at 25 ± 2 °C and 55 ± 15% humidity.

#### Anticoccidial test

A total of 120 13-day-old broilers were randomly divided into 10 groups to ensure that the initial cage weight of each group was almost the same, with 12 broilers in each group. All groups except the healthy control group (HCG) received 5 × 10^4^ oocysts orally at age 14 days. The EUC group was provided with feed to which eucalyptus oil had been added (0.20 g eucalyptus oil per 1 kg feed), for 9 days (13–22 days old). The API group was provided with feed to which apigenin had been added (0.20 g apigenin per 1 kg feed), for 9 days (13–22 days old). For the two essential oil combinations, the EUC&EUG group was provided with feed to which both eucalyptus oil and eugenol essential oil had been added (0.20 g eucalyptus oil + 0.20 g eugenol essential oil, at ratio of 1:1, per 1 kg feed), for 9 days (13–22 days old); the EUC&API group was provided with feed to which both eucalyptus oil and apigenin had been added (0.20 g eucalyptus oil + 0.20 g apigenin, at a ratio of 1:1, per 1 kg feed), for 9 days (13–22 days old); and the EUG&API group was provided with feed to which eugenol essential oil and apigenin were added (0.20 g eugenol essential oil + 0.20 g apigenin, at a ratio of 1:1, per 1 kg feed), for 9 days (13–22 days old). For the three essential oil combination, the EUC&API&EUG group was provided with feed to which 0.20 g eucalyptus oil, 0.20 g apigenin and 0.20 g eugenol essential oil per 1 kg feed (1:1:1) had been added (or weaker combinations, see section Gradient-dose (high-, medium-, and low-dose) anticoccidial tests), for 9 days (13–22 days old). The HC received no *Eimeria tenella* challenge and no treatment. The decoquinate solution group (DS) was administered to chickens by adding 1 ml decoquinate solution per 1 l drinking water for 7 days (14–21 days old). The infection control group (IC) was infected with *Eimeria tenella* but was not treated (Table 1).

**Table 1:** Botanical natural product treatments for the 10 groups

Groups	Botanical natural products or essential oils	Method of administration	Dosage of oral oocysts
EUG	Eugenol essential oil	Feed with 0.20 g/kg of eugenol essential oil for 9 days	5 × 10^4^
EUC	Eucalyptus oil	Feed with 0.10 g/kg of eucalyptus oil for 9 days	5 × 10^4^
API	Apigenin	Feed with 0.20 g/kg of apigenin for 9 days	5 × 10^4^
EUC&EUG	Eucalyptus oil + eugenol essential oil	Feed with 0.20 g/kg of eucalyptus oil + eugenol essential oil for 9 days	5 × 10^4^
EUC&API	Eucalyptus oil + apigenin	Feed with 0.20 g/kg of eucalyptus oil + apigenin for 9 days	5 × 10^4^
EUG&API	Eugenol essential oil + apigenin	Feed with 0.20 g/kg of eugenol essential oil + apigenin for 9 days	5 × 10^4^
EUC&API&EUG	Eucalyptus oil + apigenin + eugenol essential oil	Feed with 0.20 g/kg of eucalyptus oil + apigenin + eugenol essential oil for 9 days	5 × 10^4^
DS	Decoquinate solution	Feed with 1 ml/l of decoquinate solution for 7 days	5 × 10^4^
HC	–	–	–
IC	–	–	5 × 10^4^

*API* Apigenin group, *DS* decoquinate solution group, *EUC* eucalyptus oil group, *EUG* eugenol essential oil group, *HC* healthy control group, *IC* infection control group

Dietary and water status, clinical signs and mortality of broilers in each group were observed and recorded daily on day 0 post-infection, and the survival rate at the age of 22 days was calculated. When the broilers were 22 days old, all broilers were weighed one by one to calculate the relative weight gain (rBWG) rate. After the broilers were euthanized by injecting excess phenobarbital intravenously, the cecum of each group was collected, and the cecal lesions were scored [24]. The number of oocysts per gram of feces (OPG) and the oocyst index (OI) were determined from the feces on the eighth day following infection using the McMaster’s technique [17, 25]. The OI values were converted as follows: oocyst index value = 0 at a oocyst ratio of 0–1%; oocyst index value = 5 at oocyst ratio of 2–25%; oocyst index value = 10 at oocyst ratio of 26–50%; and oocyst index value = 20 at oocyst ratio of 51–75%. The following formulas were used to determine the oocyst ratio, rBWG rate, Lesion index (LI) and ACI:

- Oocyst ratio (%) = (oocyst rate in treatment groups)/(oocyst rate in the IC group) × 100% [17];
- rBWG rate (%) = (average weight gain of infection groups/average weight gain of healthy group) × 100% [18];
- LI = lesion score × 10 [18];
- ACI = (rBWG + survival rate) × 100 − (lesion score + OI value) [26].

#### Gradient-dose (high-, medium-, and low-dose) anticoccidial tests

A total of 120 13-day-old broilers were randomly divided into six groups. The initial cage weight of each group was almost the same, with 20 chickens in each group. All groups, with the exception of the HCG, received 5 × 10^4^ oocysts orally at the age of 14 days. The high-dose group (HDG) received feed treated by adding 0.20 g eucalyptus oil, 0.20 g apigenin, and 0.20 g eugenol essential oil (1:1:1) per 1 kg feed, for 9 days (13–22 days old). The medium-dose group (MDG) received feed treated with the same natural plant product combination formulation (1:1:1) at the dose of 0.l0 g of each natural product per 1 kg feed, and the low-dose group (LDG) received feed treated with the same natural plant product combination formulation (1:1:1) at a dose of 0.05 g per 1 kg feed. The HC underwent no *Eimeria tenella* challenge and no treatment. The DS was treated for 7 days (14–21 days old) by adding 1 ml decoquinate solution to 1 l drinking water, and the IC was infected with *Eimeria tenella* but was not treated (Table 2).

**Table 2:** Broiler treatments for gradient-dose anticoccidial tests

Groups	Botanical natural products or essential oils	Method of administration	Dosage of oral oocysts
HDG	Eucalyptus oil + apigenin + eugenol essential oil	Feed with 0.20 g/kg of eucalyptus oil + apigenin + eugenol essential oil for 9 days	5 × 10^4^
MDG	Eucalyptus oil + apigenin + eugenol essential oil	Feed with 0.10 g/kg of eucalyptus oil + apigenin + eugenol essential oil for 9 days	5 × 10^4^
LDG	Eucalyptus oil + apigenin + eugenol essential oil	Feed with 0.05 g/kg of eucalyptus oil + apigenin + eugenol essential oil for 9 days	5 × 10^4^
DSG	Decoquinate solution	Feed with 1 ml/l of decoquinate solution for 7 days	5 × 10^4^
HCG	–	–	–
ICG	–	–	5 × 10^4^

*DSG* Decoquinate solution group, *HCG* healthy control group, *HDG* high-dose group,* ICG* infection control group, *LDG* low-dose group, *MDG* medium-dose group

The clinical symptoms of the broilers in each group were observed and recorded. All 22-day-old broilers were weighed one by one. After euthanization, the cecum of broiler in each group was collected, and the cecal lesions were scored. The OPG, rBWG, LI, OI, and ACI were calculated as described above.

#### Safety dose test

A total of 80 14-day-old broilers were randomly divided into eight groups. The initial cage weights of each group were almost the same, with 10 broilers in each group. The dose gradient test was designed following the *Guidelines for Target Animal Safety Tests of Veterinary Chinese Medicines and Natural Medicines* [27]. The G1a group was not treated with natural plant products or chemical drugs; the G2a group received feed treated by adding 0.05 g eucalyptus oil, 0.05 g apigenin and 0.05 g eugenol essential oil (1:1:1) per 1 kg feed, for 7 days (14–21 days old); the G3a group received feed treated by adding 0.15 g eucalyptus oil, 0.15 g apigenin and 0.15 g eugenol essential oil (1:1:1) per 1 kg feed, for 7 days (14–21 days old); the G4a groups received feed treated by adding 0.30 g eucalyptus oil, 0.30 g apigenin and 0.30 g eugenol essential oil (1:1:1) per 1 kg feed, for 7 days (14–21 days old); and the G1b group was not treated with natural plant products or chemical drugs. The G2b, G3b and G4b groups received feed treated by adding eucalyptus oil, apigenin and eugenol essential oil per 1 kg feed for 14 days (14–28 days old) at doses of 0.05, 0.15 and 0.30 g at a mixture ratio of 1:1:1, respectively (Table 3).

**Table 3:** Botanical natural product treatments in the safety dose test for the combination formulation containing three plant natural products

Groups	Botanical natural products or essential oils	Method of administration
G1a	–	–
G2a	Eucalyptus oil + apigenin + eugenol essential oil	Feed with 0.05 g/kg of eucalyptus oil + apigenin + eugenol essential oil for 7 days
G3a	Eucalyptus oil + apigenin + eugenol essential oil	Feed with 0.15 g/kg of eucalyptus oil + apigenin + eugenol essential oil for 7 days
G4a	Eucalyptus oil + apigenin + eugenol essential oil	Feed with 0.30 g/kg of eucalyptus oil + apigenin + eugenol essential oil for 7 days
G1b	–	–
G2b	Eucalyptus oil + apigenin + eugenol essential oil	Feed with 0.05 g/kg of eucalyptus oil + apigenin + eugenol essential oil for 14 days
G3b	Eucalyptus oil + apigenin + eugenol essential oil	Feed with 0.15 g/kg of eucalyptus oil + apigenin + eugenol essential oil for 14 days
G4b	Eucalyptus oil + apigenin + eugenol essential oil	Feed with 0.30 g/kg of eucalyptus oil + apigenin + eugenol essential oil for 14 days

The broilers in the G1a, G2a, G3a and G4a groups were raised to the age of 21 days and weighed, following which blood was collected; the chickens were then euthanized. The broilers in the G1b, G2b, G3b and G4b groups were raised to the age of 28 days and weighed, following which their blood was collected; the chickens were then euthanized. The major organs (heart, liver, spleen, lungs, and kidneys) were weighed to calculate the relative organ weight (ROW) using the following formula: ROW (%) = organ weight (g)/body weight (g) × 100% [28].

Liver tissue and kidney tissue from each group of broilers (21 and 28 days old) were stained with hematoxylin/eosin for histopathological examination [29]. The total feed consumption was calculated and combined with body weight to calculate feed conversion ratio (FCR) and body weight gain (BWG). BWG was calculated as the difference between the mean initial body weight (on day 14) and the mean final body weight (on day 21 and 28). The FCR was calculated as the ratio of feed consumption to BWG [30].

Six broilers at 21 and 28 days of age were randomly selected and blood was collected from each group. The collected blood was subjected to five routine blood tests and the serum was subjected to six serum biochemical analyses. The blood was collected by cardiac puncture and stored in test tubes containing ethylene diamine tetraacetic acid (EDTA). Conventional hematological parameters and standard serum biochemical indicators were measured on a fully automatic blood analyzer (BC-2800vet; Shenzhen Qiaosheng Medical Technology Co., Ltd., Shenzhen, China) and a fully automatic blood biochemical detector (Chemray 240 and 800; Radiodetection Life Sciences Limited, Shenzhen, China), respectively. White blood cells (WBC), red blood cells (RBC), hemoglobin concentration (HGB), hematocrit (HCT, %), mean corpuscular hemoglobin concentration (MCHC), alanine aminotransferase (ALT), aspartate aminotransferase (AST), total protein (TP), total bilirubin (TBIL), blood urea nitrogen (BUN), creatinine (CRE) and other blood indicators were detected [31].

### Statistical analysis

The one-way analysis of variance (ANOVA) and Duncan’s multiple range test were performed to determine statistical differences between groups using GraphPad Prism 8.0 (GraphPad Software Inc., La Jolla, CA, USA).

## Results

### Anticoccidial effect of groups and single and double plant natural products

**Fig. 1:**
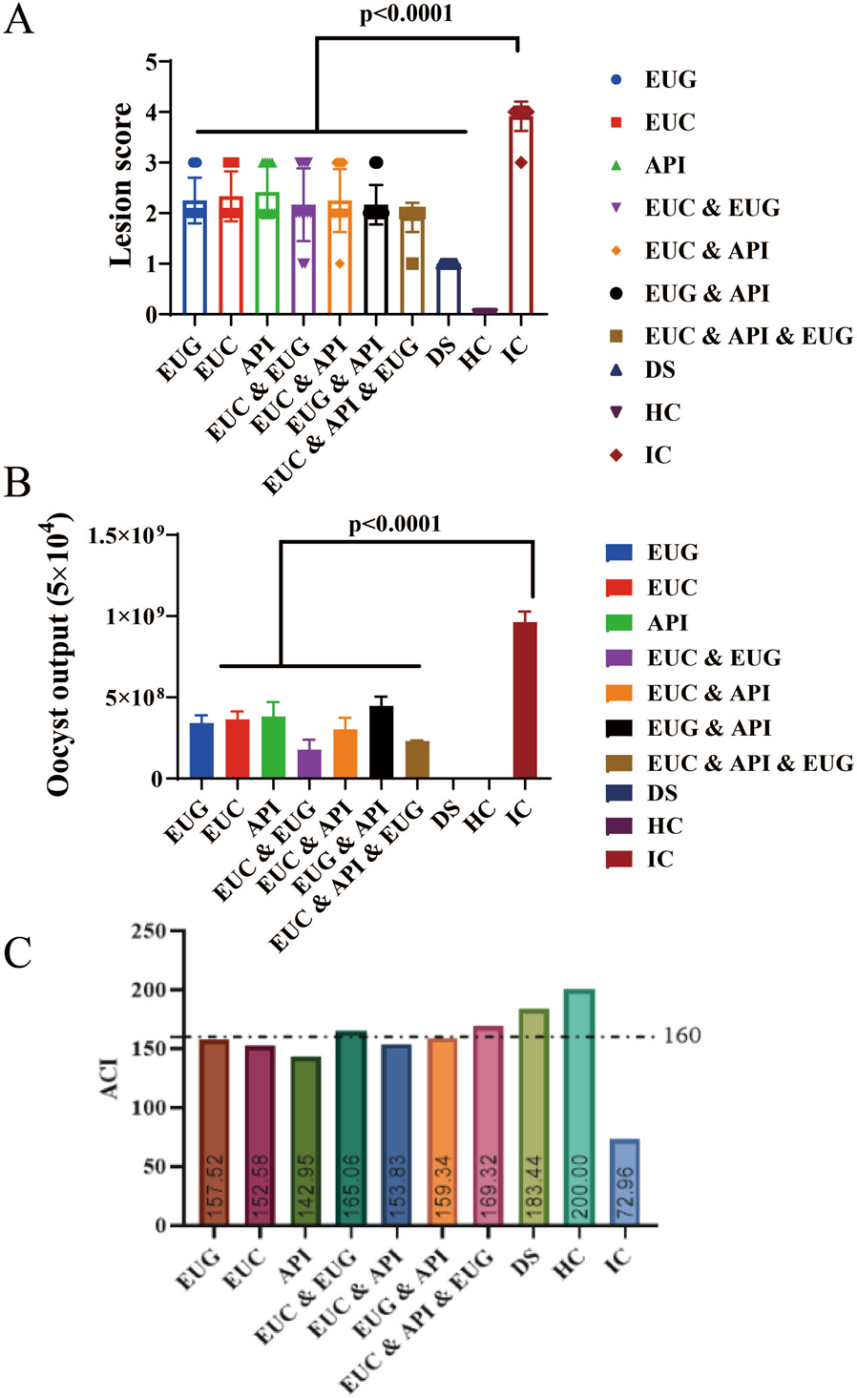
Comparison of the anticoccidial effect of the combination formulation of 3 natural plant products and of single and double plant natural products. **A** Cecal lesion scores of the combination formulation and of single and dual plant natural product groups. **B** Oocyst output (8 days post-infection) for the combination formulation and for single and dual plant natural product groups. **C** Anticoccidial index (*ACI*) of the combination formulation and of the single and dual botanical natural product groups. API, Apigenin group; DS, decoquinate solution group; EUC, eucalyptus oil group; EUG, eugenol essential oil group; HC, healthy control group; IC, infection control group

In order to evaluate the effect of the combination formulation on the weight gain of broilers, we calculated the rBWG rate (Additional file 1: Table S1). The results showed that the rBWG rate of chickens receiving the combination formulation was 93.5%, which was slightly higher than the rBWG of 93.4% for the decoquinate group, indicating the desirable weight gain effect of this formulation(Table 4). The results showed that this combination formulation had lower cecal lesions and lower oocyst output than the single- and double-component formula of plant natural products (Fig. 1a, b) (Additional file 1: Table S2, S3). In addition, the ACI of this combination formulation was 169.3, suggesting a good anticoccidial effect (Fig. 1c)(Additional file 1: Table S4).

**Table 4:** Relative weight gain rate of chickens receiving single and double plant natural products and the combination formulation of three natural plant products

Groups	Initial cage weight (g)	Final cage weight (g)	Weight gain (g)	rBWG (%)
EUG	2009	5249	3240	85.0
EUC	2009	5283	3274	85.9
API	2008	4947	2939	77.1
EUC&EUG	2012	5508	3496	91.7
EUC&API	2011	5301	3290	86.3
EUG&API	2011	5289	3278	86.0
EUC&API&EUG	2011	5574	3563	93.5
DS	2008	5569	3561	93.4
HC	2011	5822	3811	100.0
IC	2010	4314	2304	60.5

*API* apigenin group, *DS* decoquinate solution group, *EUC* eucalyptus oil group,*EUG* eugenol essential oil group, *HC* healthy control group, *IC* infection control group,* rBWG* relative body weight gain

### Anticoccidial effect of gradient doses of the combination formulation

**Fig. 2:**
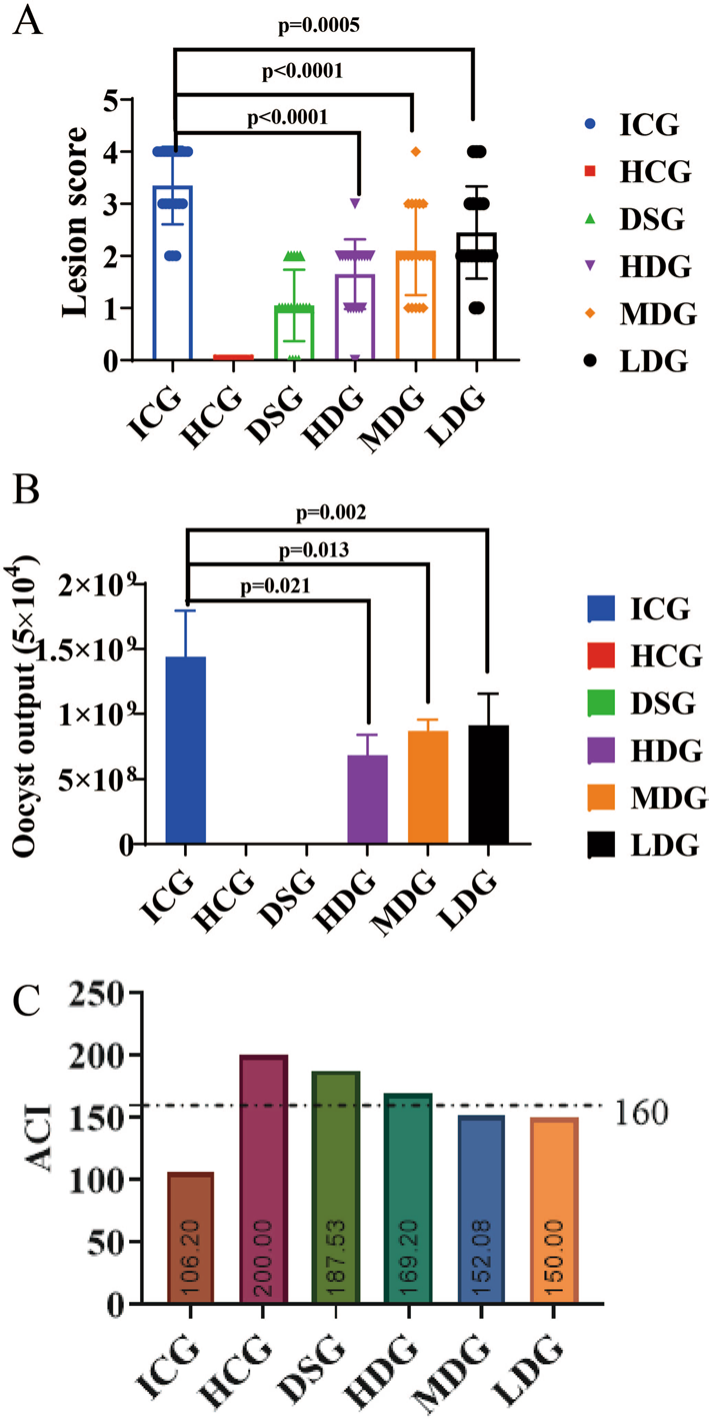
Anticoccidial effects of the high-, medium- and low-dose groups receiving the combination formulation containing 3 plant natural products. **A** Cecal lesion scores of the high-, medium- and low-dose groups. **B** Oocyst output (8 days post-infection) of the high-, medium-, and low-dose groups. **C** Anticoccidial index (ACI) of the high-, medium- and low-dose groups. DSG, Decoquinate solution group; HCG, healthy control group; HDG, high-dose group; ICG, infection control group; LDG, low-dose group; MDG, middle-dose group

In order to explore the optimal dose of the combination formulation of three plant natural products, we designed and investigated the effects of three gradient doses. The results showed that the rBWG of the high-dose group was 95.7%, which was the highest among the three dose groups, indicating that the high-dose formulation had the best weight gain effect (Table 5) (Additional file 1: Table S5). In addition, the high-dose group exhibited a lower cecal lesion score and a lower oocyst output than the mid- and low-dose groups (Fig. 2a, b) (Additional file 1: Table S6, S7). Further, we calculated the ACI, and found that the ACI of the combination formulation was 169.2, implying its good anticoccidial effect (Fig. 2c) (Additional file 1: Table S8).

**Table 5:** Relative body weight gain (rBWG) for high-, medium-, and low- dose group of the combination formulation containing 3 plant natural products

Groups	Initial cage weight (g)	Final cage weight (g)	Weight gain (g)	rBWG (%)
LDG	3317	8504	5187	94.5
MDG	3318	8427	5109	93.1
HDG	3318	8571	5253	95.7
HCG	3318	8807	5489	100.0
DSG	3319	8700	5381	98.0
ICG	3312	7687	4375	79.7

*DSG* Decoquinate solution group, *HCG* healthy control group, *HDG* high- dose group, *ICG* infection control group *LDG* low- dose group, *MDG* medium- dose group,* rBWG* relative body weight gain

### Safety dose assessment

**Fig. 3:**
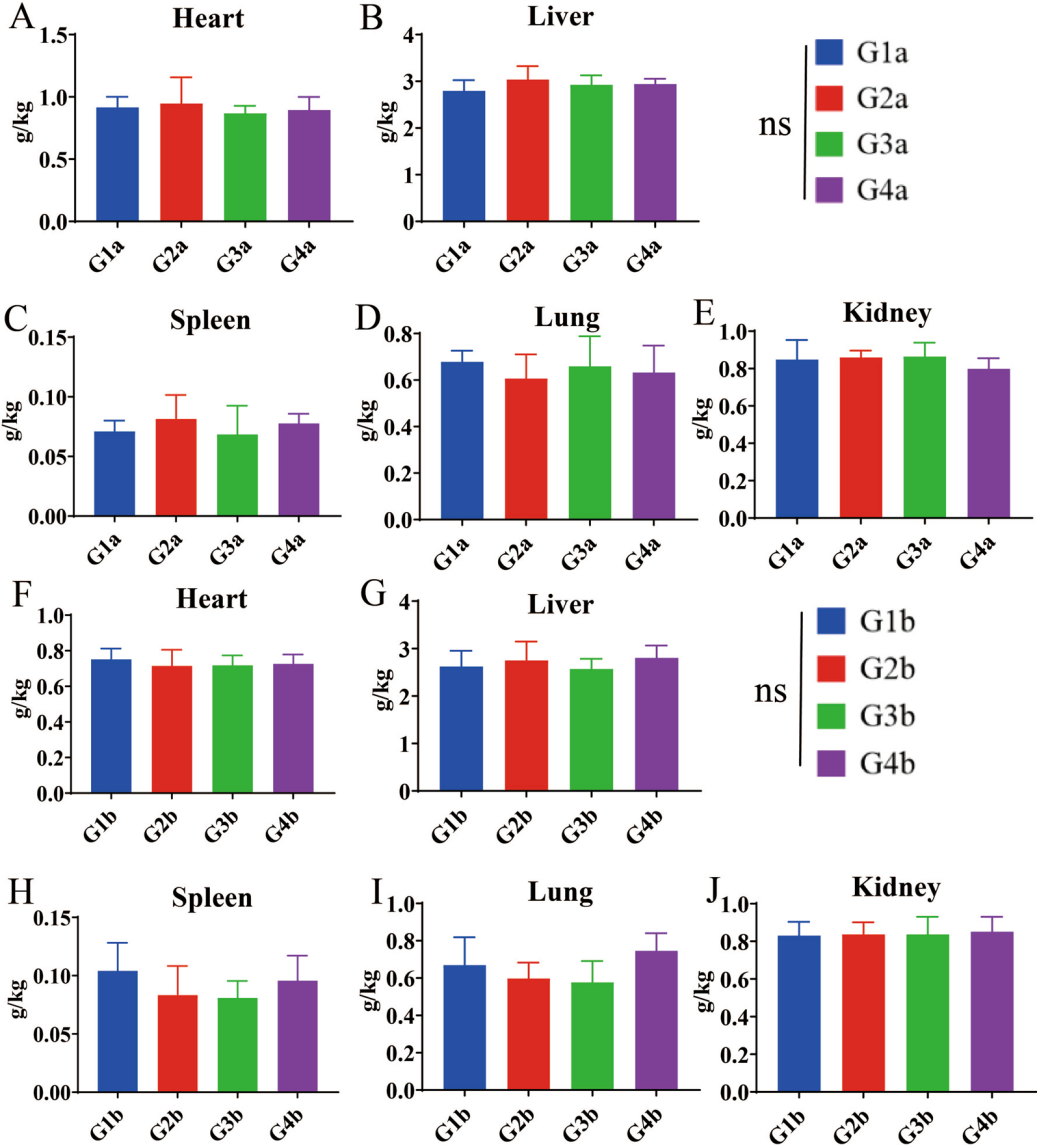
Effect of the combination formulation with 3 plant natural products on the relative organ weight (ROW) of chickens in the safety test (*n* = 6).** A**–**E** ROW of heart (**A**), liver (**B**), spleen (**C**), lungs (**D**) and kidneys (**E**) of each group after 7 days starting the combination formulation treatment.** F**–**J** ROW of heart (**F**), liver (**G**), spleen (**H**), lungs (**I**) and kidneys (**J**) of each group after 14 days starting the combination formulation treatment. See Table 3 for description of groups. ns, Not significant

**Fig. 4:**
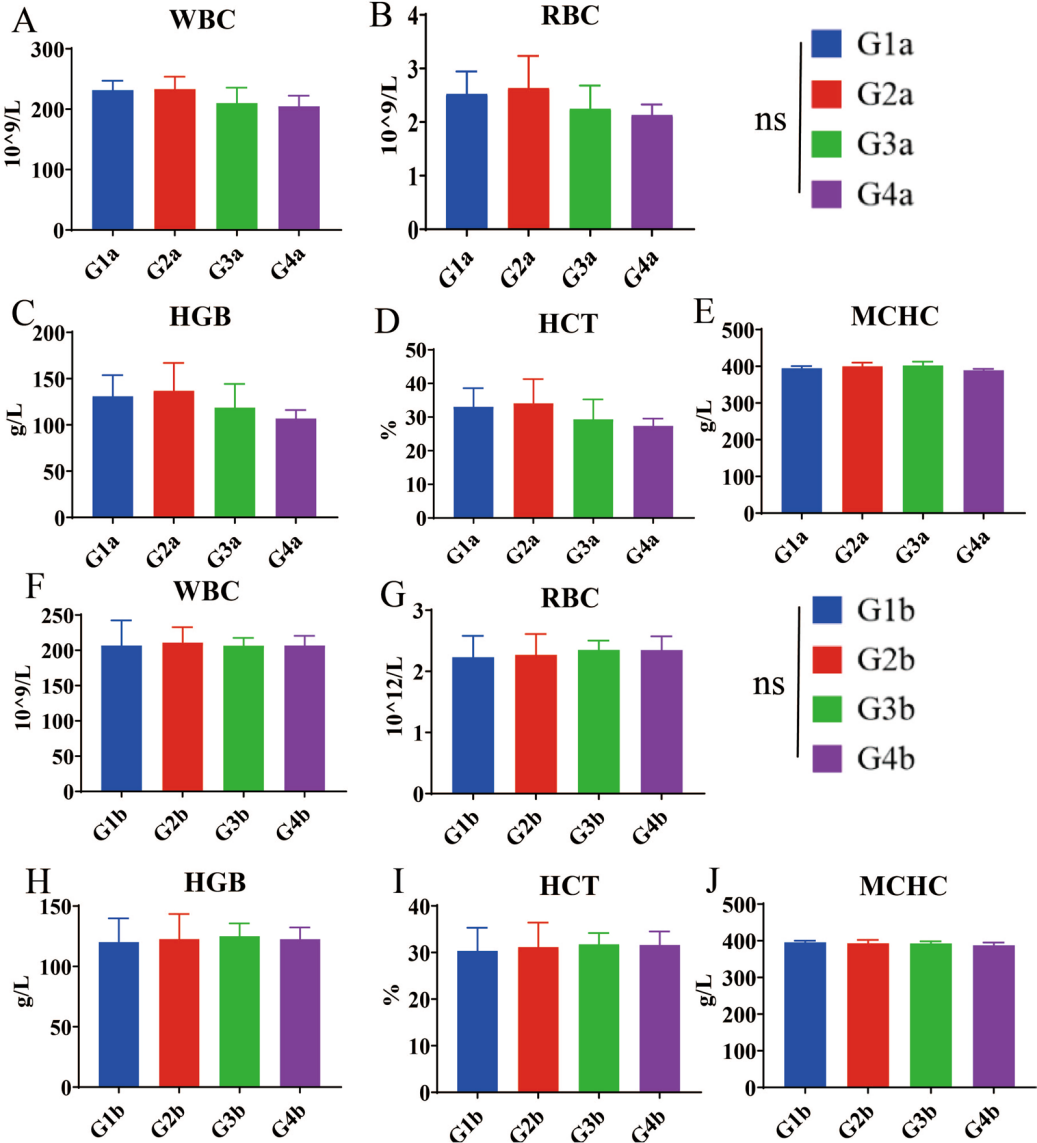
Safety dose assessment in terms of 5 indicators of hematology.** A**–**E** WBC (**A**), RBC (**B**), HGB (**C**), HCT (**D**) and MCHC (**E**) were assessed at 7 days after starting the combination formulation treatment.** F**–**J** WBC (**F**), RBC (**G**), HGB (**H**), HCT (**I**) and MCHC (**J**) were assessed at 14 days after starting the combination formulation treatment. See Table 3 for description of groups. HCT, Hematocrit; HGB, hemoglobin; MCHC, mean corpuscular hemoglobin concentration; ns, not significant; RBC, red blood cells; WBC, white blood cells

**Fig. 5:**
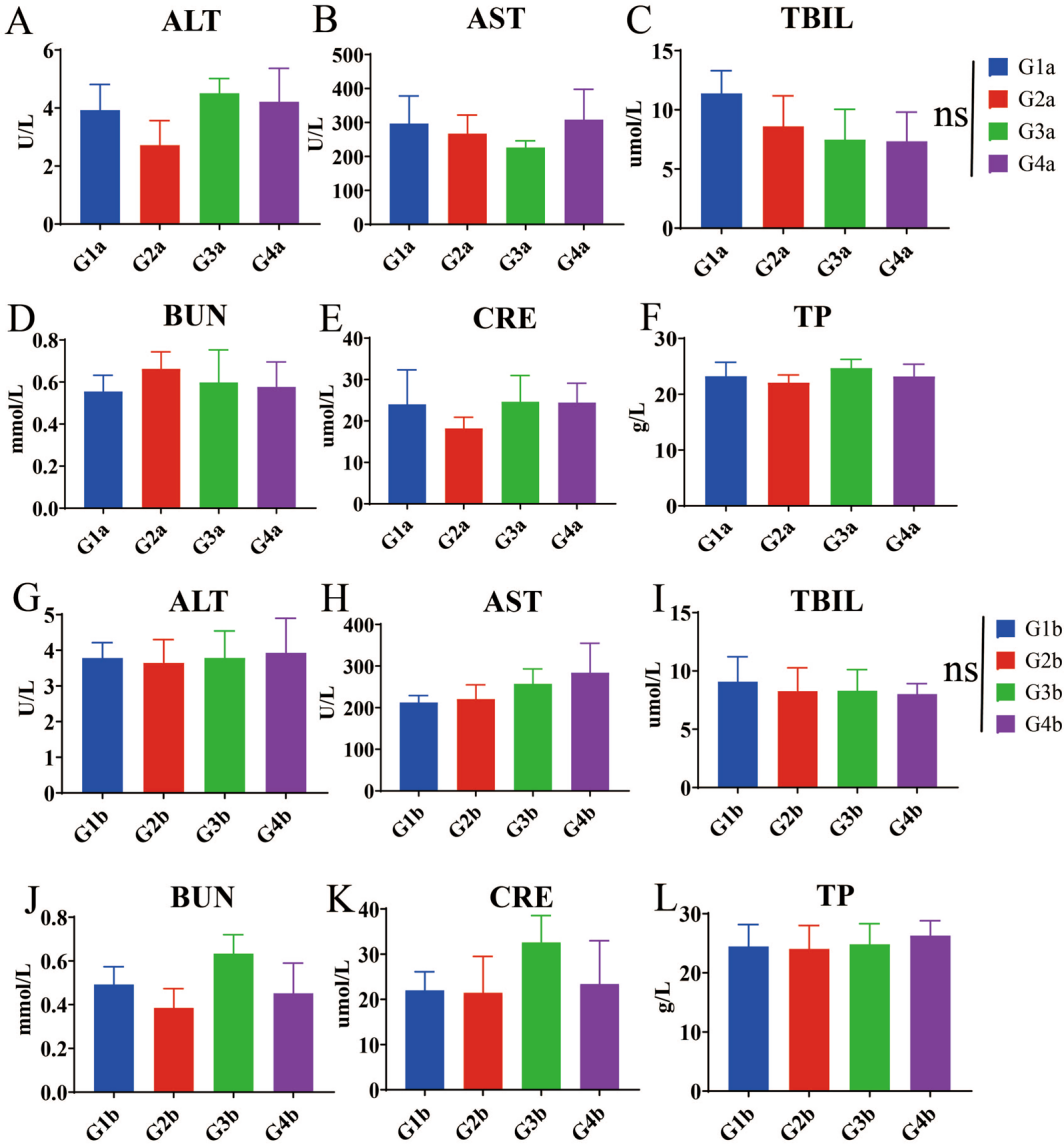
Safety dose assessment in terms of 6 indicators of serum biochemistry.** A**–**F** Detection of serum biochemical indices, including ALT (**A**), AST (**B**), TBIL (**C**), BUN (**D**), CRE (**E**) and TP (**F**), 7 days after starting the combination formulation treatment.** G**–**I** Detection of serum biochemical indices, including ALT (**G**), AST (**H**), TBIL (**I**), BUN (**J**), CRE (**K**) and TP (**L**), 14 days after starting the combination formulation treatment. See Table 3 for description of groups. ALT, Alanine aminotransferase; AST, aspartate aminotransferase; BUN, blood urea nitrogen; CRE, creatinine; ns, not significant; TBIL, total bilirubin; TP, total protein

**Fig. 6:**
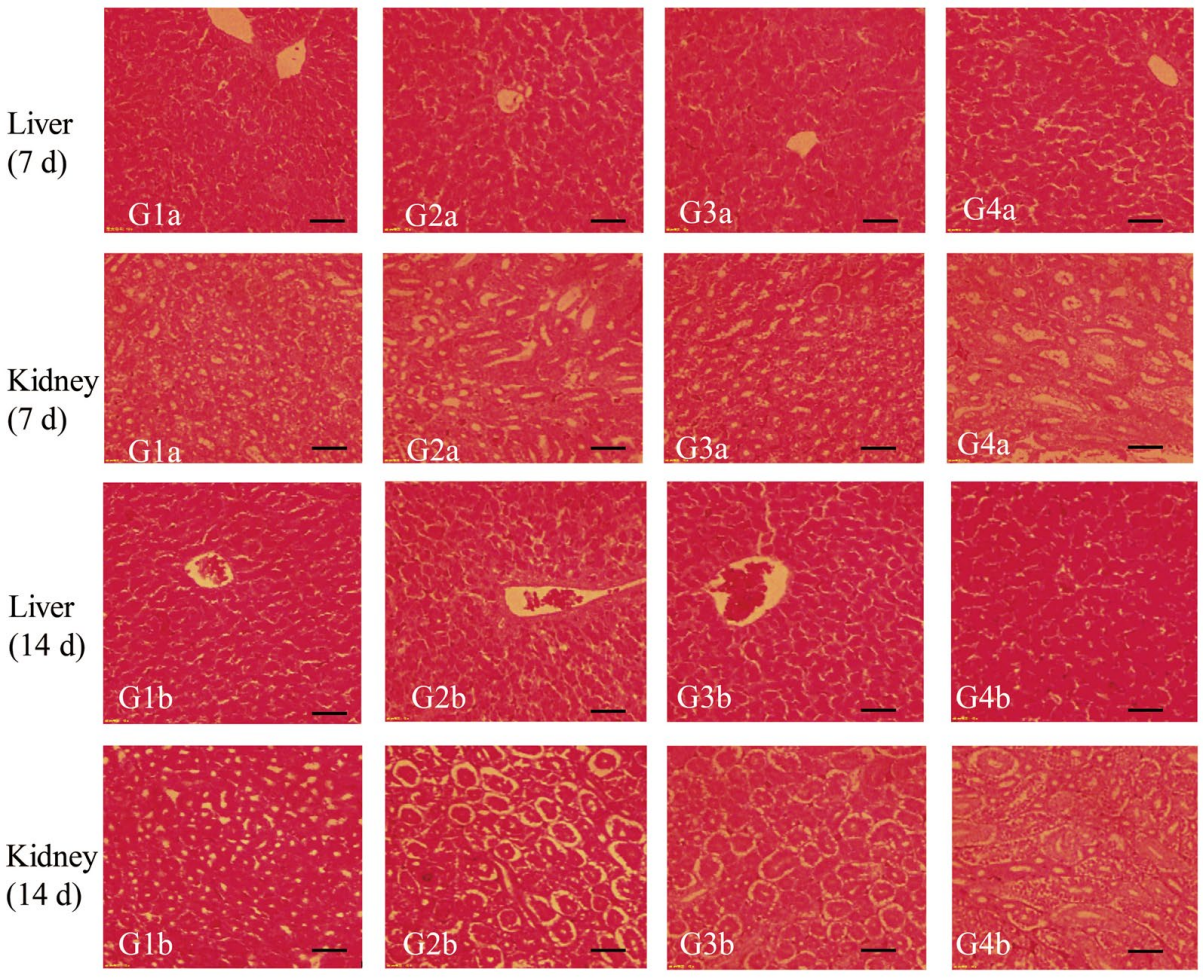
The top 2 rows show the histopathological analysis of organs (livers, 200× and kidneys, 200×) in the control group (G4a) and in 3 combination formulation-treated groups (G1a, G2a, G3a), 7 days after administration (H&E staining). Scale bar: 50 µm. The bottom 2 rows show the histopathological analysis of organs (livers, 200× and kidneys, 200×) in the control group (G4b) and in 3 combination formulation-treated groups (G1b, G2b, G3b) 14  days after administration (H&E staining). Scale bar: 50 µm. See Table 3 for description of groups. H&E, Hematoxylin and eosin stain

In order to accurately evaluate the safety of combination formulation, we designed gradient doses according to the *Guidelines for Target Animal Safety Test of Veterinary Chinese Medicines and Natural Drugs* [27]. The results showed that after 7 days of treatment with the combination formulation, the maximum FCR (1.816) was for the group treated with threefold the recommended dose (RD) (middle-dose group), compared with that for the groups treated with one- and sixfold the RD. After 14 days of treatment, the FCR in the group receiving threefold the RD also showed the highest FCR (1.617) (Table 6) (Additional file 1: Table S9). These results indicated that this combination formulation could effectively improve the FCR. We collected the main organs of broilers in the 7- and 14-day groups, and then calculated the relative organ weight (ROW). The results showed that on 7 or 14 days post-treatment, there were no significant differences in the ROW of the heart, liver, spleen, lungs and kidneys between the combination formulation treatment groups and the control groups (G1a or G1b) (Fig. 3) (Additional file 1: Table S10). Similarly, serum biochemical indicators and blood routine indicators were not significantly different between the combination formulation treatment groups and control groups (G1a or G1b) (Figs. 4, 5) (Additional file 1: Table S11, S12). In addition, no pathological damage was found in the liver or kidney sections in all treatment groups (Fig. 6). These results indicated that the combination formulation of three plant natural products was very safe.

**Table 6:** Safety dose assessment in terms of feed conversion ratio and relative body weight gain for all groups

Groups^a^	Initial cage weight (g)	Final cage weight (g)	Weight gain (g)	Intake (g)	FCR	rBWG (%)
G1a	1670	3544	1874	3438	1.834	100.0
G2a	1670	3600	1930	3569	1.849	103.0
G3a	1669	3684	2015	3660	1.816	107.5
G4a	1671	3527	1856	3444	1.856	99.0
G1b	1672	5829	4157	7196	1.731	100.0
G2b	1670	6455	4785	8046	1.682	115.1
G3b	1671	6115	4444	7184	1.617	106.9
G4b	1671	6187	4516	7760	1.718	108.6

*FCR* Food conversion rate,* rBWG* relative body weight gain

^a^See Table 3 for description of groups

## Discussion

Chicken coccidiosis seriously damages the development of the poultry industry [32, 33]. In recent years, chemical drugs used to prevent and control chicken coccidiosis have exhibited varying degrees of reduced anticoccidial effects [34]. Some researchers have proposed to replace antibiotics with natural products. For example, high concentrations of cinnamon have been found to have a moderate anticoccidial activity (ACI = 146) [35], and good anticoccidial efficacy has been reported for 10 g/kg of Fructus Meliae toosendan extracts, with an ACI of 162.56 [36]. It has also been reported that oregano essential oil was able to improve the degree of cecal lesions and reduce oocyst output after coccidia infection [37]. In this context, we have investigated whether a combination of plant-derived natural products offers superior anticoccidial effects compared to individual plant-derived products. To our knowledge, this study represents the first attempt to systematically evaluate the efficacy and safety of a plant natural product combination formulation against coccidiosis. Specifically, the evaluation was conducted from three aspects: (i) comparison of the anticoccidial effect between the combination formulation and single or double plant natural products; (ii) exploration of the optimal anticoccidial dose of the combination formulation; and (ii) safety evaluation of this formulation.

This study evaluated the anticoccidial effects of the combination formulation and of treatment with single and combinations of two natural plant products. The results showed that this essential oil combination formulation had higher rBWG, lower cecal lesion score and lower oocyst output than the single and double treatments of natural plant products, indicating that the combination formulation achieved a better weight gain effect, lower cecal lesion severity and better oocyst reduction effect. This was further verified by the high anticoccidial comprehensive evaluation index value (ACI = 169.3), which was higher than the ACI achieved by the treatments with the single and double natural plant products. These results suggest that, in general, the combination formulation exhibited a desirable anticoccidial effect despite its slightly lower ACI (183.4) than the decoquinate solution. In addition, this formulation had the advantage of presenting no risk of veterinary drug residues.

This study also evaluated the anticoccidial effect of gradient doses of the combination formulation. The results found that the high-dose group had a better weight gain effect than the low- and medium-dose groups, and that the high dose effectively alleviated the cecal lesion damage caused by *Eimeria tenella* infection and exhibited a better oocyst output reduction effect. At the same time, the ACI of the high-dose group (169.2) was higher than that of the low- and medium-dose groups, and its high ACI (> 160.0) suggested its good anticoccidial effect. It has been reported that artemisinin exerts anticoccidial effect by inhibiting the feed intake of broilers, resulting in relatively low rBWG [17]. Compared with artemisinin, our combination formulation showed a larger relative weight gain, indicating its advantages in terms of the economic benefits of broiler meat production [17]. The ACI of garlic essential oil (0.06 ml/l) has been reported to be 167.57, which is lower than that of our combination formulation, suggesting the better anticoccidial effect of our combination formulation [18].

The data presented here on the anticoccidial activity of the combination formulation in the chicken cage test is very complete. However, to know which components of the plant natural products play an effective role in the anticoccidial effect and to determine which stage of the chicken coccidiosis the component acts on, in-depth research should be performed the combines the results of the chicken coccidiosis in vitro inhibition test. Therefore, in the future, we will further improve the purification method of chicken coccidia sporozoites and merozoites, and attempt to establish an in vitro coccidia culture platform, so as to further explore the key active ingredients of the combination formulation.

Since in the gradient dose safety evaluation test, the ACI of low dose group (LDG) of combination formula reached 150.0, which we regarded as a moderate anticoccidial effect, the concentration of the low-dose group was used as the recommended dose (RD). Further investigation using different RD (one-, three- and sixfold the RD) found that after 7 and 14 days of treatment with these gradient doses of combination formulation, the ROW, five blood routine indicators and six serum biochemical indicators of major organs (heart, liver, spleen, lung and kidney) were not significantly different between the treatment groups and control groups (G1a or G1b). Similarly, there was no obvious pathological damage in the liver and kidney sections. Taken together, these results jointly indicated that our combination formulation was non-toxic to Arbor Acres broilers and had a high safety.

The above results demonstrate the desirable anticoccidial effect and high safety of our combination formulation. This study is a preliminary study of the clinical application of the anticoccidial effect of this formulation, laying a theoretical basis for the development of new plant essential oil products. The combination formulation also provides a new alternative and supplementary method for the prevention and control of chicken coccidiosis in China.

## Conclusions

This study found that a combination formulation had a good anticoccidial effect (ACI = 169.3). The combination formulation achieved better anticoccidial effects than single and double plant natural products. The high-dose group of the combination formulation exhibited the optimal anticoccidial effect, with a ACI of 169.2. The safety evaluation revealed that this formulation was non-toxic to Arbor Acres broilers, exhibiting a high safety. Therefore, the combination formulation has the potential to become an alternative to current antibiotics for the prevention and control of chicken coccidiosis.

### Supplementary Information

**Additional file 1: Table S1.** Initial and final body weights of broilers in groups of chicken cage trials with the combination formulation and single and dual plant natural products (*n* = 12).** Table S2.** Cecum lesion scores in treatment groups such as single, double and plant natural product combination formulations (*n* = 12).** Table S3**. OI of single and double plant natural products and the combination formulation of plant natural products, etc.** Table S4.** Survival of broiler chickens with single and double plant natural products and the combination formulation (*n* = 12).** Table S5.** Initial and final body weights of broilers in the plant natural product combination formulation (high, medium and low dose groups) (n = 20).** Table S6.** Cecum lesion score (8 dpi) for the plant natural product combination formulation (high, medium and low dose groups) (*n* = 20).** Table S7.** OI of single and dual product plant natural products and the combination formulation.** Table S8.** Survival rate of broiler chickens in groups of single and double botanical natural products as well as groups of the combination formulation.** Table S9.** Evaluation of the safety of the combination formulation on the body weight of broiler chickens in each group (*n* = 10).** Table S10. **ROW of major organs in each group for safety evaluation of the combination formulation (*n* = 6).** Table S11.** Evaluation of the safety of the combination formulation and blood routine indexes of each group (*n* = 6).** Table S12.** Serum biochemical indices of the groups in the safety evaluation test of the combination formulation (*n* = 6).

## Data Availability

No datasets were generated or analysed during the current study.

## Abbreviations

ACI: Anticoccidial index
FCR: Feed conversion ratio
RD: Recommended dose
rBWG: Relative body weight gain
OPG: Oocysts per gram
OI: Oocyst index
ROW: Relative organ weight
BWG: Body weight gain
EDTA: Ethylene diamine tetraacetic acid
WBC: White blood cells
RBC: Red blood cells
HGB: Hemoglobin concentration
HCT: Hematocrit
MCHC: Mean corpuscular hemoglobin concentration
ALT: Alanine aminotransferase
AST: Aspartate aminotransferase
TP: Total protein
TBIL: Total bilirubin
BUN: Blood urea nitrogen
