# Supplementary material for: Anticoccidial activity of a botanical natural product based on eucalyptus, apigenin and eugenol against Eimeria tenella in broiler chickens
Source: Parasit Vectors. 2024 Aug 2;17:327. doi: 10.1186/s13071-024-06409-z (PMC11295687; doi:10.1186/s13071-024-06409-z)
Supplement: Supplementary file 1 — Additional file 1: Table S1. Initial and final body weights of broilers in groups of chicken cage trials with the combination formulation and single and dual plant natural products (n = 12). Table S2. Cecum lesion scores in treatment groups such as single, double and plant natural product combination formulations (n = 12). Table S3. OI of single and double plant natural products and the combination formulation of plant natural products, etc. Table S4. Survival of broiler chickens with single and double plant natural products and the combination formulation (n = 12). Table S5. Initial and final body weights of broilers in the plant natural product combination formulation (high, medium and low dose groups) (n = 20). Table S6. Cecum lesion score (8 dpi) for the plant natural product combination formulation (high, medium and low dose groups) (n = 20). Table S7. OI of single and dual product plant natural products and the combination formulation. Table S8. Survival rate of broiler chickens in groups of single and double botanical natural products as well as groups of the combination formulation. Table S9. Evaluation of the safety of the combination formulation on the body weight of broiler chickens in each group (n = 10). Table S10. ROW of major organs in each group for safety evaluation of the combination formulation (n = 6). Table S11. Evaluation of the safety of the combination formulation and blood routine indexes of each group (n = 6). Table S12. Serum biochemical indices of the groups in the safety evaluation test of the combination formulation (n = 6). [file 13071_2024_6409_MOESM1_ESM.docx]

TableS1. Initial and final body weights of broilers in groups of chicken cage trials with the combination formulation and single and dual plant natural products (n=12)

| Groups | Initial broiler weight | Final broiler weight |
| --- | --- | --- |
| EUG | 139 | 332 |
|  | 145 | 393 |
|  | 149 | 399 |
|  | 155 | 412 |
|  | 160 | 465 |
|  | 165 | 443 |
|  | 168 | 428 |
|  | 172 | 454 |
|  | 178 | 415 |
|  | 184 | 432 |
|  | 192 | 473 |
|  | 202 | 503 |
| EUC | 140 | 392 |
|  | 145 | 390 |
|  | 149 | 364 |
|  | 155 | 426 |
|  | 160 | 402 |
|  | 165 | 436 |
|  | 168 | 432 |
|  | 172 | 459 |
|  | 178 | 470 |
|  | 183 | 485 |
|  | 193 | 424 |
|  | 201 | 503 |
| API | 138 | 390 |
|  | 146 | 374 |
|  | 148 | 390 |
|  | 155 | 373 |
|  | 159 | 386 |
|  | 165 | 375 |
|  | 167 | 446 |
|  | 173 | 450 |
|  | 177 | 392 |
|  | 185 | 413 |
|  | 191 | 486 |
|  | 204 | 472 |
| EUC&EUG | 141 | 450 |
|  | 144 | 334 |
|  | 150 | 430 |
|  | 154 | 442 |
|  | 161 | 431 |
|  | 164 | 432 |
|  | 169 | 514 |
|  | 170 | 462 |
|  | 180 | 515 |
|  | 182 | 476 |
|  | 197 | 472 |
|  | 200 | 550 |
| EUC&API | 140 | 317 |
|  | 145 | 365 |
|  | 150 | 440 |
|  | 155 | 396 |
|  | 160 | 455 |
|  | 164 | 445 |
|  | 169 | 482 |
|  | 172 | 395 |
|  | 179 | 527 |
|  | 183 | 526 |
|  | 194 | 442 |
|  | 200 | 511 |
| EUG&API | 140 | 353 |
|  | 145 | 366 |
|  | 150 | 415 |
|  | 154 | 416 |
|  | 161 | 426 |
|  | 164 | 432 |
|  | 169 | 424 |
|  | 171 | 443 |
|  | 179 | 472 |
|  | 182 | 484 |
|  | 196 | 522 |
|  | 200 | 536 |
| EUC&API&EUG | 143 | 450 |
|  | 143 | 381 |
|  | 152 | 482 |
|  | 152 | 420 |
|  | 162 | 434 |
|  | 162 | 483 |
|  | 169 | 472 |
|  | 170 | 460 |
|  | 180 | 492 |
|  | 181 | 530 |
|  | 198 | 410 |
|  | 199 | 560 |
| DS | 137 | 420 |
|  | 146 | 379 |
|  | 148 | 482 |
|  | 156 | 484 |
|  | 159 | 509 |
|  | 165 | 487 |
|  | 166 | 365 |
|  | 173 | 463 |
|  | 177 | 338 |
|  | 186 | 540 |
|  | 190 | 520 |
|  | 205 | 582 |
| HC | 142 | 416 |
|  | 143 | 440 |
|  | 151 | 482 |
|  | 153 | 456 |
|  | 162 | 530 |
|  | 162 | 491 |
|  | 169 | 474 |
|  | 170 | 503 |
|  | 180 | 533 |
|  | 182 | 530 |
|  | 197 | 442 |
|  | 200 | 525 |
| IC | 136 | 342 |
|  | 147 | 334 |
|  | 148 | 317 |
|  | 157 | 335 |
|  | 158 | 348 |
|  | 165 | 349 |
|  | 165 | 341 |
|  | 174 | 390 |
|  | 176 | 420 |
|  | 188 | 334 |
|  | 190 | 399 |
|  | 206 | 405 |

TableS2. Cecum lesion scores in treatment groups such as single, double and plant natural product the combination formulation (n=12)

| Groups | Lesion score |
| --- | --- |
| EUG | 2 |
|  | 2 |
|  | 2 |
|  | 3 |
|  | 2 |
|  | 2 |
|  | 2 |
|  | 2 |
|  | 3 |
|  | 3 |
|  | 2 |
|  | 2 |
| EUC | 3 |
|  | 2 |
|  | 2 |
|  | 3 |
|  | 2 |
|  | 2 |
|  | 2 |
|  | 2 |
|  | 3 |
|  | 2 |
|  | 3 |
|  | 2 |
| API | 2 |
|  | 2 |
|  | 2 |
|  | 2 |
|  | 3 |
|  | 2 |
|  | 2 |
|  | 3 |
|  | 2 |
|  | 3 |
|  | 3 |
|  | 3 |
| EUC&EUG | 2 |
|  | 1 |
|  | 2 |
|  | 2 |
|  | 3 |
|  | 2 |
|  | 2 |
|  | 2 |
|  | 3 |
|  | 1 |
|  | 3 |
|  | 3 |
| EUC&API | 2 |
|  | 3 |
|  | 3 |
|  | 2 |
|  | 2 |
|  | 2 |
|  | 3 |
|  | 2 |
|  | 2 |
|  | 1 |
|  | 3 |
|  | 2 |
| EUG&API | 2 |
|  | 2 |
|  | 3 |
|  | 2 |
|  | 2 |
|  | 2 |
|  | 2 |
|  | 2 |
|  | 2 |
|  | 2 |
|  | 3 |
|  | 2 |
| EUC&API&EUG | 2 |
|  | 2 |
|  | 2 |
|  | 2 |
|  | 2 |
|  | 2 |
|  | 2 |
|  | 2 |
|  | 2 |
|  | 2 |
|  | 1 |
|  | 2 |
| DS | 1 |
|  | 1 |
|  | 1 |
|  | 1 |
|  | 1 |
|  | 1 |
|  | 1 |
|  | 1 |
|  | 1 |
|  | 1 |
|  | 1 |
|  | 1 |
| HC | 0 |
|  | 0 |
|  | 0 |
|  | 0 |
|  | 0 |
|  | 0 |
|  | 0 |
|  | 0 |
|  | 0 |
|  | 0 |
|  | 0 |
|  | 0 |
| IC | 3 |
|  | 4 |
|  | 4 |
|  | 4 |
|  | 4 |
|  | 4 |
|  | 4 |
|  | 4 |
|  | 4 |
|  | 4 |
|  | 4 |
|  | 4 |

TableS3. OI of single and double plant natural products and the combination formulation of plant natural products.

| Groups | Number of oocysts in the counting chamber | Faecal weight (8 dpi) | Total output of the oocyst | Mean total oocyst output (8 dpi) | Relative oocyst reduction rate (%) | OI |
| --- | --- | --- | --- | --- | --- | --- |
| EUG | 1710 | 1022 | 393470000 | 342335933.3 | 35.48% | 10 |
|  | 2140 |  |  |  |  |  |
|  | 1259 |  | 298321800 |  |  |  |
|  | 1660 |  |  |  |  |  |
|  | 1800 |  | 335216000 |  |  |  |
|  | 1480 |  |  |  |  |  |
| EUC | 1390 | 1299 | 419577000 | 364586000 | 37.79% | 10 |
|  | 1840 |  |  |  |  |  |
|  | 1060 |  | 340338000 |  |  |  |
|  | 1560 |  |  |  |  |  |
|  | 1510 |  | 333843000 |  |  |  |
|  | 1060 |  |  |  |  |  |
| API | 2030 | 1090 | 427280000 | 381500000 | 39.54% | 10 |
|  | 1890 |  |  |  |  |  |
|  | 1690 |  | 439270000 |  |  |  |
|  | 2340 |  |  |  |  |  |
|  | 1180 |  | 277950000 |  |  |  |
|  | 1370 |  |  |  |  |  |
| EUC&EUG | 830 | 1025 | 227550000 | 177325000 | 18.38% | 5 |
|  | 1390 |  |  |  |  |  |
|  | 530 |  | 108650000 |  |  |  |
|  | 530 |  |  |  |  |  |
|  | 690 |  | 195775000 |  |  |  |
|  | 1220 |  |  |  |  |  |
| EUC&API | 1520 | 1052 | 358732000 | 302625333.3 | 31.37% | 10 |
|  | 1890 |  |  |  |  |  |
|  | 1830 |  | 327172000 |  |  |  |
|  | 1280 |  |  |  |  |  |
|  | 890 |  | 221972000 |  |  |  |
|  | 1220 |  |  |  |  |  |
| EUG&API | 2160 | 1203 | 486012000 | 446714000 | 46.30% | 10 |
|  | 1880 |  |  |  |  |  |
|  | 1840 |  | 381351000 |  |  |  |
|  | 1330 |  |  |  |  |  |
|  | 1710 |  | 472779000 |  |  |  |
|  | 2220 |  |  |  |  |  |
| EUC&API&EUG | 1230 | 962 | 234728000 | 230238666.7 | 23.86% | 5 |
|  | 1210 |  |  |  |  |  |
|  | 1080 |  | 226070000 |  |  |  |
|  | 1270 |  |  |  |  |  |
|  | 1360 |  | 229918000 |  |  |  |
|  | 1030 |  |  |  |  |  |
| DS | 0 | 987 | 0 | 0 | 0.00% | 0 |
|  | 0 |  |  |  |  |  |
|  | 0 |  | 0 |  |  |  |
|  | 0 |  |  |  |  |  |
|  | 0 |  | 0 |  |  |  |
|  | 0 |  |  |  |  |  |
| HC | 0 | 1209 | 0 | 0 | 0.00% | 0 |
|  | 0 |  |  |  |  |  |
|  | 0 |  | 0 |  |  |  |
|  | 0 |  |  |  |  |  |
|  | 0 |  | 0 |  |  |  |
|  | 0 |  |  |  |  |  |
| IC | 5940 | 837 | 1013607000 | 964782000 | 100.00% | 40 |
|  | 6170 |  |  |  |  |  |
|  | 7150 |  | 991008000 |  |  |  |
|  | 4690 |  |  |  |  |  |
|  | 5290 |  | 889731000 |  |  |  |
|  | 5340 |  |  |  |  |  |

TableS4. Survival of broiler chickens with single and double plant natural products and the combination formulation (n=12)

| Groups | Number of surviving broilers | Total number of broilers | Survival rate（%） |
| --- | --- | --- | --- |
| EUG | 12 | 12 | 100.00% |
| EUC | 12 | 12 | 100.00% |
| API | 12 | 12 | 100.00% |
| EUC&EUG | 12 | 12 | 100.00% |
| EUC&API | 12 | 12 | 100.00% |
| EUG&API | 12 | 12 | 100.00% |
| EUC&API&EUG | 12 | 12 | 100.00% |
| DS | 12 | 12 | 100.00% |
| HC | 12 | 12 | 100.00% |
| IC | 11 | 12 | 91.67% |

Table S5. Initial and final body weights of broilers in the plant natural product combination formulation (high, medium and low dose groups) (n=20)

| Groups | Initial weight(g) | Final weight(g) |
| --- | --- | --- |
| LDG | 146 | 250 |
|  | 148 | 314 |
|  | 150 | 307 |
|  | 153 | 335 |
|  | 154 | 460 |
|  | 157 | 413 |
|  | 158 | 383 |
|  | 160 | 465 |
|  | 163 | 449 |
|  | 165 | 460 |
|  | 167 | 421 |
|  | 169 | 475 |
|  | 170 | 428 |
|  | 172 | 470 |
|  | 173 | 444 |
|  | 175 | 466 |
|  | 178 | 482 |
|  | 182 | 501 |
|  | 187 | 496 |
|  | 190 | 485 |
| MDG | 147 | 419 |
|  | 147 | 364 |
|  | 151 | 418 |
|  | 153 | 360 |
|  | 155 | 455 |
|  | 156 | 407 |
|  | 159 | 403 |
|  | 160 | 427 |
|  | 163 | 377 |
|  | 164 | 398 |
|  | 168 | 430 |
|  | 169 | 413 |
|  | 171 | 393 |
|  | 172 | 426 |
|  | 173 | 377 |
|  | 174 | 419 |
|  | 180 | 499 |
|  | 181 | 519 |
|  | 187 | 507 |
|  | 188 | 416 |
| HDG | 147 | 411 |
|  | 147 | 308 |
|  | 152 | 364 |
|  | 152 | 348 |
|  | 155 | 412 |
|  | 156 | 360 |
|  | 159 | 438 |
|  | 160 | 448 |
|  | 164 | 436 |
|  | 164 | 478 |
|  | 168 | 443 |
|  | 169 | 399 |
|  | 171 | 401 |
|  | 172 | 426 |
|  | 173 | 465 |
|  | 173 | 490 |
|  | 180 | 426 |
|  | 181 | 529 |
|  | 187 | 485 |
|  | 188 | 504 |
| HCG | 146 | 405 |
|  | 148 | 356 |
|  | 151 | 426 |
|  | 153 | 442 |
|  | 154 | 375 |
|  | 157 | 427 |
|  | 158 | 448 |
|  | 160 | 367 |
|  | 163 | 456 |
|  | 165 | 408 |
|  | 167 | 447 |
|  | 169 | 449 |
|  | 170 | 468 |
|  | 172 | 406 |
|  | 173 | 483 |
|  | 175 | 534 |
|  | 179 | 486 |
|  | 181 | 494 |
|  | 187 | 413 |
|  | 190 | 517 |
| DSG | 146 | 372 |
|  | 148 | 359 |
|  | 151 | 378 |
|  | 153 | 381 |
|  | 154 | 368 |
|  | 156 | 445 |
|  | 159 | 466 |
|  | 160 | 377 |
|  | 163 | 417 |
|  | 165 | 438 |
|  | 168 | 481 |
|  | 169 | 478 |
|  | 171 | 434 |
|  | 172 | 440 |
|  | 173 | 459 |
|  | 174 | 437 |
|  | 179 | 485 |
|  | 181 | 488 |
|  | 187 | 517 |
|  | 190 | 480 |
| ICG | 146 | 350 |
|  | 149 | 401 |
|  | 150 | 249 |
|  | 154 | 331 |
|  | 154 | 370 |
|  | 157 | 317 |
|  | 158 | 377 |
|  | 161 | 357 |
|  | 161 | 405 |
|  | 165 | 404 |
|  | 166 | 402 |
|  | 169 | 359 |
|  | 170 | 421 |
|  | 172 | 381 |
|  | 172 | 432 |
|  | 175 | 397 |
|  | 176 | 445 |
|  | 183 | 392 |
|  | 183 | 436 |
|  | 191 | 461 |

Table S6. Cecum lesion score (8 dpi) for the plant natural product combination formulation (high, medium and low dose groups) (n=20)

| Groups | Cecum lesion score |
| --- | --- |
| LDG | 1 |
|  | 2 |
|  | 1 |
|  | 3 |
|  | 2 |
|  | 3 |
|  | 4 |
|  | 2 |
|  | 3 |
|  | 4 |
|  | 2 |
|  | 4 |
|  | 2 |
|  | 2 |
|  | 2 |
|  | 2 |
|  | 2 |
|  | 2 |
|  | 3 |
|  | 3 |
| MDG | 1 |
|  | 1 |
|  | 1 |
|  | 1 |
|  | 2 |
|  | 1 |
|  | 2 |
|  | 3 |
|  | 2 |
|  | 2 |
|  | 3 |
|  | 2 |
|  | 2 |
|  | 3 |
|  | 3 |
|  | 4 |
|  | 3 |
|  | 2 |
|  | 2 |
|  | 2 |
| HDG | 2 |
|  | 1 |
|  | 2 |
|  | 2 |
|  | 0 |
|  | 1 |
|  | 2 |
|  | 2 |
|  | 1 |
|  | 2 |
|  | 2 |
|  | 2 |
|  | 1 |
|  | 1 |
|  | 2 |
|  | 2 |
|  | 2 |
|  | 2 |
|  | 1 |
|  | 3 |
| HCG | 0 |
|  | 0 |
|  | 0 |
|  | 0 |
|  | 0 |
|  | 0 |
|  | 0 |
|  | 0 |
|  | 0 |
|  | 0 |
|  | 0 |
|  | 0 |
|  | 0 |
|  | 0 |
|  | 0 |
|  | 0 |
|  | 0 |
|  | 0 |
|  | 0 |
|  | 0 |
| DSG | 2 |
|  | 1 |
|  | 0 |
|  | 0 |
|  | 1 |
|  | 1 |
|  | 2 |
|  | 1 |
|  | 1 |
|  | 1 |
|  | 2 |
|  | 1 |
|  | 2 |
|  | 1 |
|  | 2 |
|  | 0 |
|  | 0 |
|  | 1 |
|  | 1 |
|  | 1 |
| ICG | 4 |
|  | 4 |
|  | 3 |
|  | 4 |
|  | 2 |
|  | 3 |
|  | 4 |
|  | 3 |
|  | 3 |
|  | 3 |
|  | 3 |
|  | 4 |
|  | 4 |
|  | 2 |
|  | 2 |
|  | 4 |
|  | 4 |
|  | 4 |
|  | 3 |
|  | 4 |

Table S7. OI of single and dual product plant natural products and the combination formulation

| Groups | Number of oocysts in the counting room | Faecal weight (8 dpi) (g) | Total output of the oocyst (8dpi) | Percentage relative reduction in oocysts (%) | oocyst value |
| --- | --- | --- | --- | --- | --- |
| LDG | 5535 | 1113 | 1190353500 | 63.35% | 20 |
|  | 5160 |  |  |  |  |
|  | 3672 |  | 809373600 |  |  |
|  | 3600 |  |  |  |  |
|  | 3276 |  | 734134800 |  |  |
|  | 3320 |  |  |  |  |
| MDG | 3850 | 1139 | 823610900 | 60.14% | 20 |
|  | 3381 |  |  |  |  |
|  | 3645 |  | 806412000 |  |  |
|  | 3435 |  |  |  |  |
|  | 3872 |  | 964960800 |  |  |
|  | 4600 |  |  |  |  |
| HDG | 3570 | 980 | 722358000 | 47.52% | 10 |
|  | 3801 |  |  |  |  |
|  | 4180 |  | 817320000 |  |  |
|  | 4160 |  |  |  |  |
|  | 1936 |  | 510972000 |  |  |
|  | 3278 |  |  |  |  |
| DSG | 0 | 990 | 0 | 0.00% | 0 |
|  | 0 |  |  |  |  |
|  | 0 |  | 0 |  |  |
|  | 0 |  |  |  |  |
|  | 0 |  | 0 |  |  |
|  | 0 |  |  |  |  |
| HCG | 0 | 1299 | 0 | 0.00% | 0 |
|  | 0 |  |  |  |  |
|  | 0 |  | 0 |  |  |
|  | 0 |  |  |  |  |
|  | 0 |  | 0 |  |  |
|  | 0 |  |  |  |  |
| ICG | 5670 | 959 | 1097575500 | 100.00% | 40 |
|  | 5775 |  |  |  |  |
|  | 10290 |  | 1803879000 |  |  |
|  | 8520 |  |  |  |  |
|  | 6858 |  | 1413757800 |  |  |
|  | 7884 |  |  |  |  |

Table S8. Survival rate of broiler chickens in groups of single and double botanical natural products as well the combination formulation

| Groups | Number of surviving broilers | Total number of broilers | Survival rate |
| --- | --- | --- | --- |
| LDG | 20 | 20 | 100% |
| MDG | 20 | 20 | 100% |
| HDG | 20 | 20 | 100% |
| DSG | 20 | 20 | 100% |
| HCG | 20 | 20 | 100% |
| ICG | 20 | 20 | 100% |

Table S9. Evaluation of the safety of the combination formulation on the body weight of broiler chickens in each group（n=10）

| Groups | Initial weight(g) | Final weight(g) |
| --- | --- | --- |
| G1a | 146 | 314 |
|  | 149 | 319 |
|  | 156 | 293 |
|  | 158 | 340 |
|  | 168 | 369 |
|  | 170 | 369 |
|  | 174 | 383 |
|  | 175 | 385 |
|  | 185 | 400 |
|  | 189 | 372 |
| G2a | 144 | 322 |
|  | 153 | 334 |
|  | 154 | 315 |
|  | 161 | 343 |
|  | 163 | 349 |
|  | 172 | 366 |
|  | 173 | 383 |
|  | 179 | 376 |
|  | 180 | 416 |
|  | 191 | 396 |
| G3a | 145 | 307 |
|  | 151 | 345 |
|  | 155 | 311 |
|  | 161 | 381 |
|  | 164 | 337 |
|  | 172 | 384 |
|  | 173 | 377 |
|  | 177 | 404 |
|  | 181 | 425 |
|  | 190 | 413 |
| G4a | 146 | 299 |
|  | 150 | 285 |
|  | 156 | 321 |
|  | 160 | 340 |
|  | 165 | 375 |
|  | 170 | 371 |
|  | 174 | 382 |
|  | 176 | 374 |
|  | 184 | 385 |
|  | 190 | 395 |
| G1b | 148 | 533 |
|  | 148 | 491 |
|  | 156 | 552 |
|  | 157 | 627 |
|  | 168 | 541 |
|  | 169 | 464 |
|  | 174 | 633 |
|  | 175 | 598 |
|  | 188 | 730 |
|  | 189 | 660 |
| G2b | 144 | 501 |
|  | 152 | 590 |
|  | 155 | 670 |
|  | 161 | 661 |
|  | 164 | 693 |
|  | 172 | 630 |
|  | 173 | 689 |
|  | 178 | 585 |
|  | 180 | 626 |
|  | 191 | 810 |
| G3b | 146 | 543 |
|  | 151 | 469 |
|  | 155 | 618 |
|  | 161 | 586 |
|  | 165 | 567 |
|  | 171 | 742 |
|  | 173 | 601 |
|  | 177 | 667 |
|  | 182 | 698 |
|  | 190 | 624 |
| G4b | 146 | 603 |
|  | 149 | 537 |
|  | 156 | 579 |
|  | 159 | 588 |
|  | 168 | 578 |
|  | 170 | 573 |
|  | 174 | 642 |
|  | 175 | 653 |
|  | 185 | 684 |
|  | 189 | 750 |

Table S10. ROW of major organs in each group for safety evaluation of the combination formulation (n=6)

| Groups | Heart | Liver | Spleen | Lung | Kidney | Body weight(g) |
| --- | --- | --- | --- | --- | --- | --- |
| G1a | 2.97 | 9.97 | 0.29 | 2.27 | 2.62 | 340.00 |
|  | 3.29 | 9.57 | 0.26 | 2.67 | 2.69 | 383.00 |
|  | 3.88 | 11.02 | 0.23 | 2.51 | 3.62 | 369.00 |
|  | 3.28 | 9.56 | 0.23 | 2.19 | 3.45 | 369.00 |
|  | 3.11 | 10.03 | 0.29 | 2.57 | 3.29 | 372.00 |
|  | 3.80 | 11.80 | 0.27 | 2.85 | 3.13 | 385.00 |
| G2a | 3.64 | 10.65 | 0.38 | 2.18 | 2.81 | 349.00 |
|  | 2.32 | 9.80 | 0.18 | 2.59 | 3.01 | 334.00 |
|  | 2.69 | 13.06 | 0.24 | 1.97 | 3.13 | 366.00 |
|  | 3.00 | 9.50 | 0.27 | 1.71 | 2.85 | 343.00 |
|  | 4.28 | 10.70 | 0.32 | 2.01 | 3.36 | 376.00 |
|  | 4.57 | 11.76 | 0.37 | 2.55 | 3.33 | 383.00 |
| G3a | 3.56 | 12.93 | 0.29 | 2.68 | 3.75 | 404.00 |
|  | 3.30 | 10.23 | 0.23 | 2.51 | 2.89 | 384.00 |
|  | 2.97 | 9.44 | 0.39 | 3.01 | 3.11 | 337.00 |
|  | 2.85 | 10.62 | 0.21 | 2.08 | 3.31 | 377.00 |
|  | 3.08 | 10.75 | 0.18 | 1.82 | 3.15 | 345.00 |
|  | 3.57 | 11.26 | 0.21 | 2.53 | 3.03 | 381.00 |
| G4a | 3.65 | 10.64 | 0.27 | 2.68 | 3.03 | 340.00 |
|  | 3.37 | 11.06 | 0.30 | 2.79 | 2.69 | 374.00 |
|  | 2.98 | 10.62 | 0.26 | 2.30 | 3.02 | 371.00 |
|  | 3.05 | 9.46 | 0.25 | 1.93 | 2.58 | 321.00 |
|  | 3.10 | 10.51 | 0.34 | 2.10 | 2.91 | 375.00 |
|  | 3.09 | 11.31 | 0.26 | 1.83 | 3.02 | 382.00 |
| G1b | 4.94 | 17.38 | 0.78 | 5.15 | 5.76 | 627.00 |
|  | 3.91 | 12.81 | 0.60 | 3.62 | 4.44 | 533.00 |
|  | 4.62 | 11.60 | 0.58 | 4.79 | 4.23 | 552.00 |
|  | 3.28 | 12.63 | 0.41 | 2.68 | 4.47 | 491.00 |
|  | 4.23 | 17.13 | 0.44 | 3.60 | 4.86 | 598.00 |
|  | 5.59 | 22.08 | 0.96 | 3.62 | 5.40 | 730.00 |
| G2b | 3.38 | 15.37 | 0.42 | 3.46 | 5.08 | 590.00 |
|  | 5.62 | 17.74 | 0.55 | 3.55 | 5.53 | 689.00 |
|  | 5.24 | 20.87 | 0.37 | 5.06 | 5.04 | 670.00 |
|  | 4.58 | 22.25 | 0.84 | 3.53 | 6.10 | 661.00 |
|  | 4.20 | 15.45 | 0.57 | 3.79 | 4.98 | 626.00 |
|  | 4.77 | 14.93 | 0.44 | 3.72 | 5.60 | 630.00 |
| G3b | 4.36 | 15.67 | 0.43 | 3.44 | 5.74 | 624.00 |
|  | 4.21 | 16.28 | 0.39 | 3.55 | 4.31 | 567.00 |
|  | 3.78 | 15.67 | 0.51 | 3.78 | 5.80 | 601.00 |
|  | 4.91 | 16.95 | 0.50 | 2.90 | 4.44 | 618.00 |
|  | 4.14 | 14.14 | 0.63 | 2.58 | 4.80 | 586.00 |
|  | 4.92 | 15.30 | 0.50 | 4.98 | 5.61 | 667.00 |
| G4b | 4.46 | 15.55 | 0.75 | 3.67 | 4.92 | 578.00 |
|  | 4.29 | 16.35 | 0.41 | 5.00 | 5.49 | 579.00 |
|  | 4.80 | 18.53 | 0.58 | 4.17 | 5.20 | 603.00 |
|  | 4.37 | 20.51 | 0.69 | 5.62 | 6.01 | 653.00 |
|  | 4.18 | 14.61 | 0.44 | 4.26 | 4.34 | 588.00 |
|  | 4.30 | 16.79 | 0.62 | 4.51 | 5.04 | 642.00 |

TableS11. Evaluation of the safety of the combination formulation and blood routine indexes of each group (n=6)

| Groups | WBC | RBC | HGB | HCT | HCMC |
| --- | --- | --- | --- | --- | --- |
| G1a | 214.20 | 2.22 | 113.00 | 28.70 | 393.00 |
|  | 226.80 | 2.09 | 108.00 | 27.20 | 397.00 |
|  | 238.50 | 2.92 | 152.00 | 37.60 | 404.00 |
|  | 250.10 | 2.89 | 151.00 | 38.10 | 396.00 |
|  | 214.40 | 2.15 | 109.00 | 28.20 | 386.00 |
|  | 245.50 | 2.91 | 152.00 | 38.60 | 393.00 |
| G2a | 214.40 | 2.10 | 108.00 | 27.70 | 389.00 |
|  | 211.20 | 2.31 | 115.00 | 29.50 | 389.00 |
|  | 229.30 | 2.56 | 134.00 | 33.40 | 401.00 |
|  | 229.70 | 2.16 | 118.00 | 28.40 | 415.00 |
|  | 254.60 | 3.09 | 162.00 | 40.30 | 401.00 |
|  | 260.80 | 3.61 | 184.00 | 45.30 | 406.00 |
| G3a | 215.00 | 2.19 | 118.00 | 28.30 | 416.00 |
|  | 228.10 | 2.50 | 137.00 | 33.60 | 407.00 |
|  | 167.80 | 1.61 | 80.00 | 20.70 | 386.00 |
|  | 198.40 | 2.06 | 105.00 | 26.40 | 397.00 |
|  | 210.00 | 2.26 | 118.00 | 29.40 | 401.00 |
|  | 241.30 | 2.89 | 154.00 | 37.80 | 407.00 |
| G4a | 175.20 | 1.82 | 90.00 | 23.50 | 382.00 |
|  | 216.70 | 2.23 | 110.00 | 28.20 | 390.00 |
|  | 210.20 | 2.27 | 114.00 | 29.00 | 393.00 |
|  | 210.20 | 2.14 | 108.00 | 27.70 | 389.00 |
|  | 223.30 | 2.34 | 115.00 | 29.50 | 389.00 |
|  | 193.20 | 2.01 | 104.00 | 26.50 | 392.00 |
| G1b | 210.10 | 2.31 | 124.00 | 31.20 | 397.00 |
|  | 213.90 | 2.32 | 123.00 | 31.20 | 394.00 |
|  | 223.70 | 2.40 | 130.00 | 33.30 | 390.00 |
|  | 224.40 | 2.41 | 129.00 | 32.80 | 393.00 |
|  | 233.00 | 2.42 | 134.00 | 33.20 | 403.00 |
|  | 136.10 | 1.52 | 81.00 | 20.40 | 397.00 |
| G2b | 214.80 | 2.53 | 133.00 | 34.60 | 384.00 |
|  | 214.00 | 2.37 | 129.00 | 33.40 | 386.00 |
|  | 228.50 | 2.39 | 135.00 | 32.90 | 410.00 |
|  | 217.70 | 2.29 | 124.00 | 31.70 | 391.00 |
|  | 223.00 | 2.45 | 134.00 | 33.80 | 396.00 |
|  | 167.90 | 1.60 | 81.00 | 20.70 | 391.00 |
| G3b | 223.20 | 2.56 | 142.00 | 35.30 | 402.00 |
|  | 204.20 | 2.31 | 120.00 | 30.50 | 393.00 |
|  | 192.90 | 2.16 | 114.00 | 29.00 | 393.00 |
|  | 203.30 | 2.29 | 121.00 | 31.20 | 387.00 |
|  | 209.30 | 2.44 | 128.00 | 32.90 | 389.00 |
|  | NA | NA | NA | NA | NA |
| G4b | 211.20 | 2.45 | 129.00 | 32.90 | 392.00 |
|  | 214.90 | 2.38 | 122.00 | 31.80 | 383.00 |
|  | 181.80 | 1.94 | 104.00 | 26.10 | 398.00 |
|  | 222.10 | 2.62 | 132.00 | 34.70 | 380.00 |
|  | 203.40 | 2.33 | 126.00 | 32.10 | 392.00 |
|  | 206.90 | 2.37 | 122.00 | 32.10 | 380.00 |

NA: not applicable.

TableS12. Serum biochemical indices of the groups in the safety evaluation test of the combination formulation (n=6)

| Groups | ALT | AST | BUN | CRE | TBIL | TP |
| --- | --- | --- | --- | --- | --- | --- |
| G1a | 3.62 | 348.63 | 0.65 | NA | 13.78 | 18.26 |
|  | 5.42 | 425.56 | 0.47 | 18.93 | 8.77 | 23.29 |
|  | 3.74 | 232.65 | 0.51 | 40.87 | 12.28 | 23.98 |
|  | 2.69 | 208.04 | 0.61 | 20.65 | 11.53 | 25.28 |
|  | 4.00 | 257.37 | 0.48 | 20.87 | 12.53 | 24.30 |
|  | 4.10 | 309.57 | 0.60 | 22.16 | 9.52 | 24.27 |
| G2a | 2.74 | 287.49 | 0.63 | 19.47 | 10.27 | 20.43 |
|  | 1.42 | 176.09 | 0.73 | 15.17 | 3.51 | 20.39 |
|  | 2.06 | 226.88 | 0.79 | 20.33 | 8.52 | 23.53 |
|  | 3.35 | 285.80 | 0.60 | 16.03 | 9.27 | 22.64 |
|  | 3.67 | 317.00 | 0.57 | 21.83 | 10.02 | 23.17 |
|  | 3.09 | 310.07 | 0.67 | 16.46 | 10.02 | 22.42 |
| G3a | 4.50 | 215.24 | 0.64 | 16.67 | 11.02 | 25.81 |
|  | 4.13 | 207.40 | 0.69 | 18.72 | 5.51 | 24.16 |
|  | 4.95 | 254.19 | 0.78 | 23.56 | 9.52 | 25.26 |
|  | 3.75 | 207.28 | NA | 26.57 | 4.01 | 24.70 |
|  | 5.09 | 239.21 | 0.48 | 33.67 | 7.02 | 26.31 |
|  | 4.66 | 235.93 | 0.40 | 28.72 | 7.78 | 21.87 |
| G4a | 5.43 | 365.69 | 0.51 | 32.05 | 4.51 | NA |
|  | 3.84 | 238.34 | NA | 26.43 | 9.27 | 21.78 |
|  | 2.46 | 454.37 | NA | 17.96 | 5.01 | 21.54 |
|  | 3.53 | 219.08 | 0.48 | 24.31 | 8.02 | 26.85 |
|  | 4.80 | 314.10 | 0.75 | 22.37 | 10.77 | 22.18 |
|  | 5.25 | 259.02 | 0.57 | 23.56 | 6.51 | 23.61 |
| G1b | 3.86 | 235.17 | 0.51 | 25.92 | 3.51 | 21.20 |
|  | 3.14 | 232.21 | 0.57 | 17.64 | 11.78 | 23.26 |
|  | 3.59 | 198.77 | 0.37 | 20.44 | 7.77 | 23.43 |
|  | 4.09 | 204.28 | 0.58 | NA | 14.28 | 31.72 |
|  | 4.38 | 199.93 | 0.43 | 19.36 | 7.02 | 24.32 |
|  | 3.65 | 205.16 | 0.50 | 26.78 | 9.77 | 22.81 |
| G2b | 3.00 | 234.20 | 0.48 | NA | 10.52 | 30.97 |
|  | 4.84 | 268.66 | 0.31 | 25.28 | 4.76 | 22.76 |
|  | 3.89 | 204.66 | 0.39 | 34.20 | 9.27 | 21.54 |
|  | 3.22 | 242.73 | 0.26 | 22.80 | 8.27 | 26.58 |
|  | 3.46 | 174.59 | 0.45 | 20.54 | 7.52 | 21.71 |
|  | 3.47 | 200.84 | 0.44 | 12.15 | 9.27 | 20.79 |
| G3b | 4.42 | 224.72 | 0.55 | 35.39 | 5.51 | 22.52 |
|  | 4.42 | 288.61 | 0.69 | 30.01 | 11.02 | 23.63 |
|  | 4.56 | 247.41 | 0.53 | 25.28 | 7.52 | 21.75 |
|  | 3.04 | 313.74 | 0.71 | 39.26 | 8.27 | 31.40 |
|  | 2.95 | 233.90 | 0.60 | 27.21 | 9.02 | 25.42 |
|  | 3.36 | 237.51 | 0.73 | 38.51 | 8.52 | 24.35 |
| G4b | 2.83 | 265.01 | 0.46 | 16.03 | 8.02 | 26.44 |
|  | 2.81 | 264.37 | 0.48 | 39.04 | 9.27 | 29.01 |
|  | 4.51 | 410.91 | 0.23 | NA | 6.77 | 24.76 |
|  | 4.47 | 238.45 | 0.39 | 25.06 | 7.52 | 28.33 |
|  | 5.18 | 211.92 | 0.50 | 27.64 | 7.77 | 27.08 |
|  | 3.79 | 313.93 | 0.65 | 20.76 | 8.77 | 22.18 |

NA: not applicable.
